# Molecular mechanisms of mitochondrial AAA+ proteases

**DOI:** 10.1016/j.jbc.2026.111264

**Published:** 2026-02-06

**Authors:** S. Quinn W. Currie, Monica M. Goncalves, Aaron D. Schimmer, Siavash Vahidi

**Affiliations:** 1 Department of Molecular and Cellular Biology, University of Guelph, Guelph, Ontario, Canada; 2 Princess Margaret Cancer Centre, University Health Network, Toronto, Ontario, Canada

**Keywords:** LONP1, ClpXP, YME1L, i-AAA, m-AAA, mitochondrial proteostasis

## Abstract

Mitochondrial AAA+ proteases, LONP1, ClpXP, YME1L (i-AAA), and the m-AAA complex, maintain protein quality and shape organelle function. Growing interest in these enzymes stems from their association with neurodegeneration, cardiomyopathy, metabolic disease, and cancer. Recent structural and biophysical work clarifies how ATP-driven conformational cycles enable substrate recognition, unfolding, translocation, and proteolysis, and how assembly state, subunit composition, and regulatory inputs tune activity. These insights help interpret patient variants and guide experiments that connect mechanism to phenotype. Here we review shared mechanistic principles across the four proteases, contrast their architectures and regulatory features, and relate these properties to substrate selection and disease mechanisms, with emphasis on evidence from structural, biochemical, and cellular studies. We also survey strategies to modulate function. Small molecules, exemplified by Dordaviprone (ONC201), which activate human ClpP, provide proof of concept, and emerging modalities such as engineered macromolecules may offer the selectivity and localization required to correct disease mechanisms or exploit disease dependencies. By integrating mechanisms, disease links, and modulation strategies, this review provides a framework for translating basic insight on mitochondrial AAA+ proteases into new tools and, ultimately, therapies.

The mitochondrion originates from the engulfment of a free-living α-proteobacterium by a eukaryotic ancestor over 1.2 billion years ago (1, 2). Unsurprisingly, mitochondria retain some of the molecular machinery of their prokaryotic ancestor, including their own genome, ribosomes, and elaborate proteolytic systems that degrade both damaged and regulatory proteins (3). These proteolytic systems are crucial for regulating organellar function (4). As up to 99% of the mitochondrial proteome (mitoproteome) is encoded by nuclear genes, intricate protein import, folding, and processing pathways are required for the generation of native mitochondrial proteins (5). The complexity of maintaining mitoproteome homeostasis is further compounded by the production of reactive oxygen species (ROS) from oxidative phosphorylation (OXPHOS). ROS impart oxidative damage on the mitoproteome, demanding innate mitochondrial protein quality control (mtPQC) mechanisms to clear damaged proteins and prevent protein aggregate accumulation (6). Additionally, fundamental mitochondrial processes, including fission and fusion (7), biogenesis (8), mitophagy (9), and apoptosis (10) are regulated by the degradation of regulatory proteins. Remodeling of the mitoproteome in response to changes in cellular metabolism similarly relies on proteolytic processing (11). Collectively, these functions are carried out by a diverse class of mitochondrial proteases (mitoproteases), which are critical in maintaining mitochondrial homeostasis (Fig. 1). The dysregulation of mitoproteases is implicated in numerous human pathologies, including cancer (12, 13, 14, 15, 16), developmental disorders (17, 18), diabetes (19), and neurodegeneration (20, 21, 22). Deterioration of mitoprotease efficiency and the associated accumulation of oxidatively damaged proteins is correlated with ageing (23, 24, 25), further highlighting the importance of mitochondrial proteostasis in human health.

Mitoproteases span a diverse group of enzymes with distinct functions. They are traditionally grouped into two classes: those that process precursor polypeptides to generate mature mitochondrial proteins, and those that maintain mtPQC by degrading damaged or misfolded proteins (10). Mitochondrial processing peptidases, comprised of an array of ATP-independent proteases, such as PMPCB, IMMP1L, IMMP2L, MIPEP (mitochondrial intermediate peptidase), and METAP1D, specifically cleave mitochondrial proteins during or shortly after import, generating mature polypeptides (26, 27). Like their prokaryotic ancestors, mitochondria lack a ubiquitin-proteasome system to mediate mtPQC (28, 29). Instead, they rely on the second functional mitoprotease class comprised of ATP-independent and ATP-dependent AAA+ (ATPases Associated with diverse cellular Activities) proteases (Fig. 1) (29). For example, ATP23 and HTRA2 operate independently of ATP and facilitate complex V assembly in *Saccharomyces cerevisiae* (30) and the degradation of oxidized proteins of the intermembrane space (IMS) (31), respectively. A network of mitochondrial AAA+ proteases, comprised of the matrix-localized LONP1 and ClpXP, and inner mitochondrial membrane (IMM) -bound i-AAA and m-AAA (Table 1), play crucial roles in the mtPQC by forming the first line of defence against mitochondrial proteotoxic stress (Fig. 1) (10). Each of these four enzymes harnesses the free energy released from ATP hydrolysis to unfold and translocate damaged and misfolded proteins into their proteolytic chambers, where substrates are degraded into short peptides.

**Figure 1:**
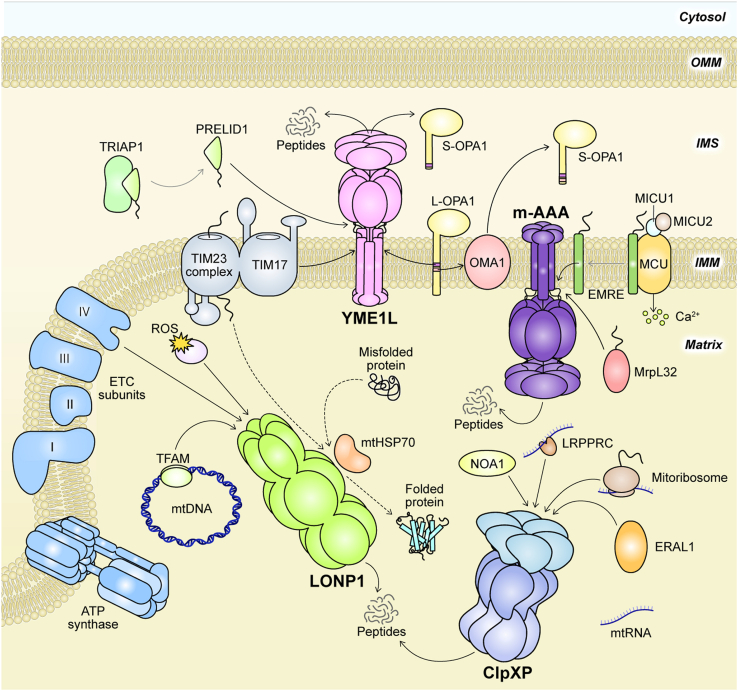
**Overview of mitochondrial regulation and PQC by AAA+ proteases**. The four human mitochondrial AAA+ proteases – LONP1, ClpXP, YME1L, and m-AAA – collectively clear damaged proteins and regulate organellular processes spanning the IMS, IMM, and matrix subcompartments. These processes include mitochondrial biogenesis, protein, lipid, and Ca^2+^ import, mitochondrial dynamics, mtDNA maintenance and expression, translation, mitophagy, and apoptosis. *Black solid lines* with arrows denote proteolytic processing. *Black dashed lines* denote chaperone-mediated folding. *Solid grey lines* denote dissociation of protein complexes.

**Table 1:** The four human mitochondrial AAA+ proteases

AAA+ protease	Protein name	UniProt Accession	Oligomeric State(s)	Protease class	Mitochondrial subcompartment	Reference
LONP1	LONP1	P36776	Homohexamer	Serine protease	Matrix	(50)
ClpXP	ClpX	O76031	Homohexamer	–	Matrix	(135)
	ClpP	Q16740	Homotetradecamer	Serine protease		(134)
i-AAA	YME1L	Q96TA2	Homohexamer	Metalloprotease	IMM-bound, IMS-facing	(35)
m-AAA	AFG3L2	Q9Y4W6	Homohexamer or heterohexamer	Metalloprotease	IMM-bound, matrix-facing	(358)
	Paraplegin	Q9UQ90	Heterohexamer			(359)

Mitochondrial AAA+ proteases themselves are highly regulated proteolytic machines, with control exerted at both the molecular and structural levels. Each one assembles into self-compartmentalizing oligomeric complexes with active sites sequestered within a proteolytic chamber to prevent spurious protein degradation (32, 33). A broadly conserved substrate unfolding and translocation mechanism arising from shared structural features housed within the AAA+ domain further controls the destruction of specific AAA+ substrates (32). For example, the AAA+ modules of each mitochondrial AAA+ protease form hexameric rings that bind ATP using a nucleotide-binding motif (Walker-A motif: GxxxxGK[T/S]), hydrolyze the γ-phosphate group *via* a Walker-B motif (hhhhDE), and depend on intersubunit contacts to facilitate around-the-ring ATP hydrolysis (34, 35, 36, 37, 38). Yet, despite these commonalities, these four proteases still maintain nuanced functional differences including mechanisms of substrate engagement, allosteric regulation driven by nucleotide and substrate binding, and association of binding partners to facilitate efficient substrate processing (35, 36, 37, 38, 39). Much of the mechanistic insight into mitochondrial AAA+ proteases is available today largely thanks to the foundational work of pioneering scientists in the field, including the late Alfred Goldberg. Dr Goldberg dedicated his career to elucidating mechanisms of protein turnover *via* AAA+ proteases and the role of intracellular protein degradation in maintaining cellular homeostasis (40, 41, 42). His contributions to the study of regulated proteolysis were transformative, including the identification and characterization of the human 26S proteasome (43) and its inhibitor, bortezomib (Velcade) (44), which remains a standard therapy for multiple myeloma (45). The Goldberg group also characterized bacterial homologues of two of the four mitochondrial AAA+ proteases—LONP1 and ClpXP (46, 47). Several recent reviews have covered key aspects of mitochondrial proteases (25, 33, 48, 49). Here, we honour some of Dr Goldberg’s contributions by reviewing both foundational and recent studies that elucidate the structure-function relationship of each of the four human mitochondrial AAA+ proteases while highlighting the structural and biophysical evidence revealing their modes of regulation and substrate processing. Emphasis will be placed on how mitochondrial AAA+ dysregulation is implicated in human health and how they can be therapeutically targeted to advance treatment options against human diseases.

## LONP1 protease

### LONP1 domain organization

**Figure 2:**
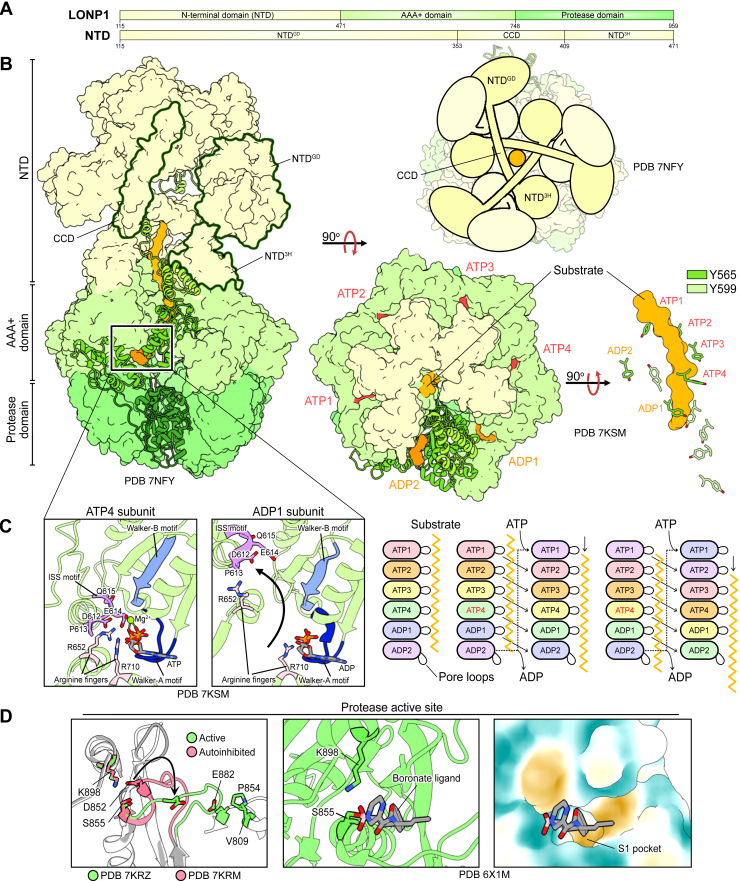
**Structural insights into the LONP1 functional mechanism**. *A*, each LONP1 polypeptide chain contains an MTS (residues 1–114) and three functional domains: an N-terminal domain (NTD); a AAA+; and a protease domain. The NTD contains an N-terminal globular subdomain (NTD^GD^), a coiled-coil subdomain (CCD), and a 3-helix subdomain (NTD^3H^). *B*, LONP1 assembles into a homohexameric, barrel-like complex, with the NTDs capping the two stacked rings formed by the AAA+ and protease domains. The NTD^GD^s form a trimer of dimers at the apical surface of the complex, with each dimer comprised of two NTD^GD^s originating from opposing subunits of the ring assembly. Conformational changes derived from ATP hydrolysis in Y565 and Y599 of pore loops 1 and 2, respectively, thread substrates unidirectionally into the chamber formed by the protease domains. *C*, around-the-ring ATP hydrolysis necessitates inter-subunit communication, as structural elements including the ISS motif and R652 arginine finger of neighbouring subunits collectively stabilize ATP in the nucleotide binding cleft. ATP hydrolysis releases these elements, eliciting structural changes in pore loops that manifest in translocation. The canonical strict hand-over-hand model summarizes the mechanism by LONP1 and other mitochondrial AAA+ proteases process substrates. *D*, substrates are processed by a Ser-Lys catalytic dyad whose activity is regulated by the structurally dynamic “catalytic loop” (residues: 844–855). Structures of LONP1 bound to peptidic inhibitors (*e*.*g*., boronate compounds) provide direct evidence of the active protease domain conformation and further reveal biophysical features of the substrate-binding pockets, thereby explaining substrate cleavage preferences.

Human mitochondrial Lon peptidase 1 (LONP1; also known as LONP, LonHS, hLON, PRSS15) is one of several members of the AAA+ superfamily regulating mtPQC. LONP1 was first identified due to its high sequence similarity to the related *Escherichia coli* Lon (*Ec*Lon), originally termed the “La protease” by Alfred Goldberg (46, 50, 51, 52). Lon proteases, including LONP1, constitute the third family within the HCLR (HslU/ClpAB/Lon/RuvB) clade of AAA+ motors (32, 53). This family is divided into bacterial and archaeal Lon proteases, with LONP1 belonging to the bacterial subgroup (53). Consistent with this classification, LONP1 largely preserves the same domain organization and functional mechanism as its bacterial homologues (37, 54, 55, 56, 57, 58). LONP1 assembles as a homohexamer and features three distinct domains (Fig. 2*A*). An N-terminal domain (NTD) caps the nearly 600-kDa barrel-shaped complex and regulates protein substrate recognition and oligomeric assembly (58, 59, 60). Two highly conserved catalytic domains comprise the core of the proteolytic complex (Fig. 2*B*). The first is a ring-shaped AAA+ domain that hydrolyzes ATP to unfold and traffic protein substrates into the proteolytic core. A C-terminal protease domain forms this proteolytic core, with a catalytic dyad comprised of conserved Lys and Ser residues essential for degradation sequestered within the barrel (54). LONP1 is encoded by a nuclear gene and is expressed with an N-terminal mitochondrial targeting sequence (MTS; residues 1–114) (58). Upon import into the matrix, the MTS is cleaved, yielding the mature form of the LONP1 polypeptide. Unlike its soluble mitochondrial matrix-localized counterpart, ClpXP, LONP1 incorporates all domains on the same polypeptide chain. Notably, a related peroxisome-specific isozyme of Lon protease (LONP2) bears similar structural architecture and degrades misfolded peroxisomal proteins (61, 62). A comprehensive review of LONP2’s role in peroxisomal function and human health can be found in Pomatto *et al*. (2016) (63).

### LONP1 function

LONP1 regulates turnover of proteins implicated in nearly every aspect of mitochondrial biology, including enzymes of the citric acid (TCA) cycle (64), glutamine biosynthesis, and steroid hormone biosynthesis pathways (65), subunits of respiratory complexes involved in OXPHOS (66), mitochondrial DNA (mtDNA) transcription factors (67), and signaling proteins regulating mitophagy (Fig. 1) (68). LONP1 also maintains mitoproteome quality by clearing damaged and misfolded proteins in the matrix (50, 66, 69). Indeed, LONP1 is upregulated in response to oxidative stress, when oxidation and damage to matrix proteins become significantly elevated (Fig. 1) (70, 71). For example, oxidative modification of ACO2 (aconitase 2), a key enzyme of the TCA cycle, exposes regions of hydrophobic patches on the protein surface (64). This promotes its engagement and degradation by LONP1, preventing the accumulation of protein aggregates (64).

Unlike the cytosolic ubiquitin-proteasome system, LONP1 lacks a strict substrate-tagging mechanism in which proteins are explicitly marked for degradation. Rather, one of the prevailing models of substrate recognition developed from studies of *Ec*Lon suggests engagement by the LONP1 NTD largely depends on unique biophysical properties associated with the misfolded or damaged state of its substrates (72, 73). Steady-state kinetics employing natively folded and denatured protein substrates reveal that *Ec*Lon engages exposed hydrophobic motifs in misfolded and damaged substrates (72, 73). It is thought that modifications of matrix proteins resulting from the induction of cellular stress, dissociation of complexes, and cleavage events expose these hydrophobic motifs that would otherwise be inaccessible in a stably folded protein (72, 73). It is apparent that sequence-specific motifs within native substrates also promote LONP1-mediated degradation. This has been most extensively characterized in bacterial homologues, where numerous degradation motifs (degrons) have been identified and characterized both *in vitro* and *in vivo*. These include the 11-residue SsrA tag (74), a recognition signal shared with ClpXP; the C-terminal residues of SulA (75), HspQ (76), and Y2853 (76); the N-terminal residues of SoxS (77) and UmuD (78); and an internal recognition motif within β-galactosidase. Both *Ec*Lon and *Yersinia pestis* Lon strongly prefer C-terminal histidine residues (79, 80). Similarly, a structure of the *Caulobacter crescentus* Lon NTD in complex with an allosterically activating adaptor, LarA, reveals that C-terminal histidine binding deep within a well-conserved NTD groove primes Lon for engagement of hydrophobic motifs of additional protein substrates (80). LONP1 and its bacterial homologues show the greatest sequence divergence in their NTDs. For example, LONP1 and *Ec*Lon exhibit only 27% sequence identity within their NTDs. This increases to 45% across the AAA+ and protease domains. Given the sequence divergence between human and bacterial Lon NTDs and the distinct proteomes they surveil, bacterial Lon substrate recognition rules may have limited translational relevance for parsing LONP1 substrate engagement mechanisms. Indeed, cross-species substrate degradation exists between bacterial homologues, while processing of these same substrates by LONP1 is significantly impaired (81).

LONP1 function extends beyond that of a protease, with additional functions that are central to mitochondrial biology, including chaperone activity. This was first established through the expression of a proteolytically inactive *S*. *cerevisiae* LONP1 homologue, Pim1 (82). The substitution of the catalytic serine, but retention of functional AAA+ domains, established that the mitochondrial Lon AAA+ function is necessary for proper folding and assembly of respiratory complexes (82). Furthermore, LONP1 is part of a broader network of mtPQC members that employ a chaperone function to prevent protein aggregation in the matrix (83, 84). LONP1 associates with mitochondrial chaperone systems, mtHSP60 and mtHSP70, the latter of which cooperates with LONP1 to facilitate the folding of newly imported protein cargo to the matrix (Fig. 1) (84, 85). Expression of LONP1 with a functional AAA+ domain, but a catalytically inactive proteolytic domain established that ATPase function, but not proteolytic function, is required for mtHSP70-LONP1-mediated folding (84), corroborating earlier Pim1 chaperone investigations (82). In the absence of mtHSP70-LONP1 chaperone system, approximately 10% of the mitoproteome aggregates, pointing to the importance of LONP1 in maintaining mitochondrial protein solubility (84). Beyond depending on mtHSP70 for protein folding, LONP1 appears to have its own intrinsic chaperone function independent of other mitochondrial chaperones (84).

LONP1 is distinct from all other mitoproteases as it maintains the integrity of the mitochondrial genome (mtDNA) through direct engagement of DNA (86, 87, 88). Like its bacterial homologues, LONP1 binds single-stranded DNA (86, 87, 88, 89). LONP1 engages GT-rich regions of the mtDNA promoter elements present on the heavy strand, particularly regions that have a propensity to form G-quadruplexes, though the LONP1 motif responsible for such is not yet known (86, 87, 90). LONP1 associates with mitochondrial nucleoids, complexes comprised of regulatory proteins engaged with mtDNA to facilitate DNA packaging, replication, transcription, or repair (91). Beyond this, LONP1 also post-translationally regulates the levels of mitochondrial transcription factor A (TFAM; Fig. 1) (67). Phosphorylation of TFAM prevents DNA binding through electrostatic repulsion, promoting its degradation by LONP1 (67). LONP1 also degrades unphosphorylated and DNA-free TFAM, thereby preventing its accumulation in the matrix (67). It is plausible that DNA-bound TFAM conceals LONP1 degrons that might otherwise be recognized in the phosphorylated, DNA-free state.

### Structural insights into LONP1 function

In the past, molecular-level understandings of LONP1 function relied on the extrapolation of crystallographic structures of its isolated domains or that of its bacterial homologues (55, 92, 93). More recently, however, LONP1 electron cryomicroscopy (cryo-EM) studies have revealed nuanced structural insights that underlie its mechanism of processive protein degradation (37, 56, 57, 58). At the apical surface of the LONP1 complex, the N-terminal globular subdomains (NTD^GD^) of the NTD assemble as a trimer of dimers, with each dimer comprised of two NTD^GD^ originating from opposing subunits of the ring assembly (37, 57, 60) (Fig. 2*B*). The coiled-coil domains (CCDs) connect the NTD^GD^ to the barrel-like core of the LONP1 complex. CCDs of alternating subunits traverse the width of the LONP1 complex and together form a triangular-shaped pore 6 Å in diameter that sits directly above the central pore formed by the AAA+ and protease domains below (37, 57, 60) (Fig. 2*B*). Each of these three CCDs contain a ratcheting finger, Y394, oriented toward the central pore that intercalates between substrate residues to prevent backsliding during translocation (37, 56, 57, 60). A 3-helix subdomain (NTD^3H^) connects the CCDs to the AAA+ ring below.

The NTD is highly dynamic and often evades structural determination in the context of the LONP1 complex (37, 56, 58). In cryo-EM studies, the NTD consistently yields the lowest resolution relative to the other domains (57, 58). Beyond its substrate engagement role, the LONP1 NTD allosterically activates the catalytic domains of LONP1 independent of ATP (60, 94, 95). Substitution of the NTD with a synthetic hexamerization domain reduces ATPase rates by 9-fold and proteolytic capacity by 17-fold, revealing the drastic activating effect that substrate engagement by the NTD has on the catalytic domains (60). This is supported by studies of *Ec*Lon that confirmed that a structurally conserved NTD loop region between the CCD and NTD^3H^ (*Ec*Lon: 239–252) is essential for ATPase and proteolytic function (94). This is particularly striking given the considerable distances between the conserved LONP1 NTD loop region and the AAA+ and proteolytic active sites, spanning approximately 55 Å and 80 Å, respectively.

In a substrate-free state, cryo-EM structures reveal LONP1 adopts a left-handed open lock-washer conformation (37). Here, ADP occupies the nucleotide-binding cleft formed a large N-terminal α/β Rossman fold subdomain and the C-terminal α-helical small subdomain, a scaffold common to all four mitochondrial AAA+ proteases (37, 96). In line with AAA+ domain activation mechanisms of *Ec*Lon, substrate engagement by the NTD^GD^ induces conformational changes that propagate through the CCD to facilitate nucleotide exchange in the AAA+ ring, starting with the subunit with the greatest helical pitch (37, 97). LONP1 structures with substrate stalled in the central channel reveal that the passage of substrates from the NTD to the AAA+ domain induces the closure of the AAA+ ring to form a right-handed spiral staircase encircling the substrate (Fig. 2*B*) (37, 56). During translocation, five of six AAA+ subunits descend in pitch, with a sixth subunit acting as the “seam” joining those with the lowest and highest pitch (Fig. 2*B*) (37). The four AAA+ subunits with the highest pitch are loaded with ATP (highest pitch: ATP1, lowest pitch: ATP4; Fig. 2*B*), while the lowest pitch domain and seam subunits retain ADP (ADP1 and ADP2, respectively; Fig. 2*B*) (37). Within the AAA+ ring, LONP1 couples conformational changes derived from ATP hydrolysis to motions of aromatic residues within pore loop 1 and pore loop 2, Y565 and Y599, respectively, to thread substrate towards the protease chamber. Mirroring the AAA+ subunit conformation, the pore loops adopt a right-handed spiral staircase conformation surrounding the vertical channel through which protein is threaded (Fig. 2*B*) (56). In all ATP-bound and the ADP1 subunits, Y565 of pore loop 1 interacts with the translocating substrate, while that of the seam subunit (ADP2) remains disengaged from the substrate (Fig. 2*B*) (37). Pore loop 2 supports translocation, with Y599 of the ATP1 and ATP2 subunits engaged with substrate (Fig. 2*B*) (37). An intersubunit signalling (ISS) motif (^612^DPEQ^615^) communicates nucleotide state between LONP1 subunits by interacting in trans with the nucleotide of the adjacent AAA+ subunit (Fig. 2*C*) (37, 56, 57). D612 stabilizes the R652 arginine finger which contacts the ATP γ-phosphate, holding the ISS element in the trans configuration (Fig. 2*C*). A second arginine finger (R710), lent by the small AAA+ subdomain of the cis LONP1 subunit, further helps stabilize the negatively charged ATP phosphate groups (Fig. 2*C*). Upon ATP hydrolysis by a water molecule activated by the catalytic glutamate of the Walker-B motif (^586^LILIDE^591^), the ISS motif and R652 arginine finger retract from the nucleotide cleft of the neighbouring subunit (Fig. 2*C*) (56).

For decades, the standard for understanding substrate translocation by AAA+ proteases, including LONP1, has followed the hand-over-hand model driven by sequential around-the-ring ATP hydrolysis, with AAA+ subunit conformation being strictly determined by nucleotide state (Fig. 2*C*) (37, 57, 97, 98, 99, 100). According to this model, ATP hydrolysis in the ATP4 subunit triggers the release of the ISS motif in the adjacent ADP1 subunit, driving a downward helical register shift throughout the AAA+ ring (Fig. 2*C*). As the ADP1 subunit moves into the seam position, its pore loops disengage from the substrate (Fig. 2*B*). The seam subunit advances up the helical register to the ATP1 position, inducing nucleotide exchange of ADP for ATP (Fig. 2*C*). Upon nucleotide exchange, the subunit’s pore loops re-engage the substrate, propelling its translocation through the central pore at a rate of 2 residues per ATP hydrolysis event (Fig. 2*C*) (32, 56). The pertinence of this strict hand-over-hand mechanism as a framework for describing AAA+ motor function has been the subject of considerable debate (101, 102, 103, 104). Indeed, the relevance of this model has more recently been called into question as it relates to the LONP1 functional cycle (60). Recent structures of LONP1 translocating substrate with all six AAA+ subunits occupied by ADP is incongruent with a strict hand-over-hand mechanism. This and other cases in the literature have led to the proposition of a more “relaxed” hand-over-hand model, where LONP1 AAA+ domains toggle between burst and dwell phases (60). Where the strict hand-over-hand model requires that ATP hydrolysis occurs in the ATP4 position, the relaxed model postulates that ATP hydrolysis events can occur concurrently, including in AAA+ subunits of higher registers (*i*.*e*. ATP1-ATP3) of the staircase (60). This manifests in a burst phase of substrate translocation *via* pore loops 1 and 2, resulting in larger steps of substrate being translocated, of up to six residues per ATP consumed.

The LONP1 protease domain switches conformations throughout the proteolytic functional cycle (37, 55, 105). Structural elements within the protease active site toggle between active and autoinhibited states (Fig. 2*D*). During substrate translocation through the AAA+ pore, like the AAA+ domain, the protease ring also adopts a right-handed spiral staircase conformation (37, 56, 57). Here, S855 of the protease dyad remains sequestered within a 3_10_ helix, with a “catalytic loop” (^844^VPEGATPKDGPS^855^) immediately preceding the catalytic serine retaining a compact conformation (37, 56, 57, 106) with D852 occluding the dyad spatial arrangement necessary for catalysis (Fig. 2*D*) (37, 56, 106). Substrate entry into the protease domain induces planar C6 symmetry, prompting protease active sites to transition to the active conformation (37, 56). Here, the catalytic loop takes on an elongated conformation, forming contacts with V809, P854, and E882 of the adjacent clockwise subunit (Fig. 2*D*) (37, 56). This structural transition sequesters D852 away from the active site, positioning the dyad in a configuration sufficient for catalysis (Fig. 2*D*) (37, 56). This regulation of protease domain conformation appears to be a conserved feature from bacterial homologues and serves as another layer of regulation to prevent spurious protein degradation in the mitochondrial matrix (105, 107).

Structures of LONP1 in complex with boronate substrate mimics reveal that protease domain activation generates a substrate binding groove around the interior of the protease ring with a hydrophobic substrate binding pocket (S1 pocket; Fig. 2*D*) (56). Likewise, mass spectrometry analysis of the peptidic products generated *in vitro* by LONP1 confirms its cleavage preference for hydrophobic residues in the P1 position (56, 81, 108). Similar investigations of *Ec*Lon and other bacterial Lon proteases including those from *Y*. *pestis* and *Mycoplasma pneumoniae*, reveal this is a conserved cleavage site preference, with a strong preference for ALFVM residues in the same position (79). Structures of substrate-trapped LONP1 (K898A) reveal that only one of the six protease subunits acts on a single polypeptide chain at a time (56). Electron density contributed by the trapped substrate appears approximately five times weaker than that of the LONP1 complex (56).

### Implications of LONP1 in human health and therapeutic potential

LONP1 is closely related to several human diseases and pathologies. CODAS, a genetic developmental disorder characterized by cerebral, ocular, dental, auricular, skeletal anomalies, is caused by mutations in *LONP1* (109, 110). These mutations are localized largely within sequences that encode the AAA+ domain, particularly at the subunit interfaces within the ring (17, 109). This disrupts cooperativity that is essential for ATP hydrolysis, prompting inefficient ATP consumption and unproductive substrate translocation (17). *In vitro* characterization of several LONP1 disease variants (P676S, R721G, S631Y, and A724V) revealed markedly reduced proteolytic activity, demonstrating the deleterious impact of CODAS-associated *LONP1* mutations (17). Notably, these variants also exhibited impaired small-peptide degradation rates, highlighting the allosteric nature of LONP1 function, wherein mutations in the AAA+ domain compromise the intrinsic activity of the distal protease domain (17).

Cancer cells have distinct biosynthetic and bioenergetic requirements that necessitate metabolic reprogramming undertaken by mitochondria, thereby imparting damage to the mitoproteome (111). In many cases, cancer cells rely on mtPQC regulators, including LONP1, to mitigate damage to the matrix proteome (13, 112, 113, 114, 115, 116). Indeed, LONP1 overexpression has been noted in several cancer types, including pancreatic, glioma, prostate, colorectal, oral, lymphoma, and cervical cancers (13, 112, 113, 114, 115, 116). LONP1 expression in cancer cells is frequently negatively correlated with patient survival (114, 116, 117). Furthermore, LONP1 expression positively correlates with cancer progression in some cases (118). While nearly absent in normal mucosa cells, LONP1 levels increase in adenomas, reaching their highest levels in colorectal cancer (118). In addition, LONP1 helps prevent senescence in skin and colorectal tumors by remodeling respiratory chain subunits to permit a transition from an oxidative to glycolytic metabolism (117). Inhibition strategies leveraging shRNA or chemical inhibitors have shown success in reducing cancer cell growth, validating LONP1 as a potential therapeutic target in cancer types that uniquely depend on it for growth and proliferation (13, 113, 114).

Given its multifunctional nature, significant efforts have focused on understanding how LONP1 mechanistically supports cancer cell survival, a question central to its potential as a therapeutic target. Several inhibitors of the LONP1 protease domain have been characterized to probe its role of clearing damaged proteins in supporting cancer cell growth (Table 2). These include obtusilactone A, (−)-sesamin (119), MG262 (120), CC4 (114), and bortezomib (121), all of which target the protease active site. Of these, obtusilactone A and (−)-sesamin have shown some promise in reducing growth and proliferation in lung cancer cells lines (119), while CC4 reduces growth in glioma cells *in vitro* (114). However, growing evidence points to the importance of the LONP1 ATPase-powered chaperone function in preventing mitochondrial protein aggregate accumulation upon the induction of oxidative stress in some cancer cell types (13, 112), necessitating inhibition strategies that extend beyond that of canonical orthosteric protease inhibitors. The sole group of LONP1 ATPase small-molecule inhibitors belongs to a class of synthetic triterpenoids comprised of bardoxolone (CDDO) and its derivatives (Table 2) (13, 122, 123, 124). These compounds inhibit both LONP1 proteolytic and chaperone functions, inducing mitochondrial protein aggregation and cell death (13, 122). Investigations into the mechanism of action of this inhibitor class have yielded contradictory findings (125). Molecular docking analysis predicted multiple binding sites for CDDO and its derivatives, and surprisingly, substitution of key amino acids within these sites led to *enhanced* rather than decreased inhibition (125). Though effective, these inhibitors bear modest affinity and have additional cellular targets beyond LONP1 (13, 126). Further structural characterization of these inhibitors to elucidate AAA+ domain regulatory hotspots, and innovative strategies predicated on disrupting LONP1 ATPase function, such as development of conventional and non-conventional effectors that prevent assembly of this obligate oligomeric enzyme, are of great therapeutic interest.

**Table 2:** Known small-molecule inhibitors and activators of the four human mitochondrial AAA+ proteases

AAA+ protease	Small-molecule inhibitors		Small-molecule activators		References
	Orthosteric	Allosteric	Orthosteric	Allosteric	
LONP1	obtusilactone A, (−)-sesamin, MG262, CC4, bortezomib	CDDO, CDDO derivatives	–	–	(13, 114, 119, 120, 121, 122, 123, 124)
ClpXP	α-aminoboronic compounds^a^, β-lactones, phenyl esters	–	α-aminoboronic compounds^a^	ONC201, ADEPs, D9, ZK53	(12, 39, 205, 224, 225, 226)
i-AAA	–	–	–	–	–
m-AAA	–	–	–	–	–

a Exhibits hormesis, where low concentrations activate and high concentrations inhibit activity.

## ClpXP protease

### ClpXP domain organization

Mitochondrial ClpXP is a proteolytic complex formed *via* the association of the ClpX unfoldase (127, 128) and the ClpP protease (129, 130). The ClpXP complex was first recognized in *E*. *coli*, shortly after the paralogue ClpAP had been identified as protease Ti by Alfred Goldberg (47, 131, 132). The human ClpX and ClpP orthologues were later characterized as nuclear-encoded proteins expressed with an MTS (residues 1–56) (128, 133). Following import into the mitochondrial matrix, the MTS is cleaved to generate the mature forms of ClpX and ClpP. Within the matrix, ClpP relies exclusively on ClpX for chaperone activity (128, 133). Human ClpP assembles as a stacked pair of homoheptameric rings forming a 340 kDa barrel-like proteolytic chamber (134). Human ClpX forms a 380 kDa homohexameric ring and binds ClpP coaxially to create a continuous channel to the degradation chamber (135, 136). Substrate degradation relies on the coordinated interplay between ClpX and ClpP, which is regulated allosterically (137).

### ClpXP function

ClpXP overexpression is linked to mitochondrial unfolded protein response (UPR^mt^) regulation in mammals (138, 139, 140) and in *C*. *elegans* (141, 142, 143). UPR^mt^ is triggered by the accumulation of mitochondrial precursor proteins and increased cytosolic ROS levels, leading to the upregulation of stress-response genes, including mitochondrial chaperones and proteases (144, 145). The upregulation of ClpXP alleviates proteotoxic stress by degrading aberrantly folded proteins and restoring mitochondrial proteostasis (10, 41, 146). The majority of mitochondrial ClpXP substrates or interactors participate in oxidative phosphorylation and mitochondrial translation (Fig. 1) (147, 148, 149, 150). These include respiratory chain components such as complex I and II subunits (151, 152, 153), and ATP synthase subunits (149). Nitric oxide-associated protein-1 (NOA1) is a matrix GTPase that modulates respiratory chain activity in response to oxygen concentrations, and is degraded by ClpXP (Fig. 1) (154, 155). ClpXP regulates mitochondrial translation by degrading proteins involved in post-transcriptional processing of mtRNA, including ERAL1 and LRPPRC, as well as ribosome-stalled mitochondrial peptides (Fig. 1) (156, 157, 158). Additional substrates of ClpXP include members of the TCA cycle, fatty acid, and amino acid metabolic pathways (68, 159, 160, 161, 162).

While ClpP alone degrades only small peptide substrates, associating with ClpX enables processive protein degradation (163, 164). Upon recognizing a labeled substrate, ClpX begins to unfold and translocate the polypeptide into the ClpP protease for degradation (165, 166). This mechanism is evolutionarily conserved and has been extensively studied in bacterial homologues (167). In Gram-negative bacteria, a co-translationally added SsrA degron (AANDENYALAA in *E*. *coli*) marks incompletely synthesized polypeptides for degradation by ClpXP or ClpAP, with recognition facilitated by adaptor proteins (168, 169, 170, 171). In Gram-positive bacteria, like *Bacillus subtilis*, an additional unfoldase, ClpC, associates with ClpP and, guided by adaptor proteins, targets substrates with phosphorylated arginine residues (172, 173). We recently found that human mitochondrial ClpX recognizes substrates bearing phosphorylated serine or threonine residues (174), but this is unlikely to be the only mode of substrate recognition by the complex. Polymerase δ-interacting protein (POLDIP2) is a human ClpXP adaptor protein that protects ClpX from LONP1-mediated degradation and facilitates substrate recognition (175, 176). POLDIP2 is proposed to direct the heme biosynthetic enzyme aminolevulinic acid synthase (ALAS) to ClpX for degradation by ClpXP (177). ClpP-independent functions for mitochondrial ClpX have also been reported in heme biosynthesis *via* activation of ALAS (178). ClpX binds and unfolds a substructure of ALAS, gating access of the pyridoxal 5′-phosphate (PLP) cofactor to the active site (179). ClpX functions beyond ClpP-mediated proteolysis have also been implicated in mtDNA maintenance by enhancing the DNA-binding activity of TFAM, thereby promoting the distribution of mtDNA nucleoids (180, 181). In human fibroblasts, ClpX has been reported to interact with fatty acid oxidation (FAO) -related proteins and its knockout enhances FAO without altering the expression levels of these interactors (182). This suggests an independent chaperone role for ClpX in FAO, although the underlying mechanism remains unclear (182).

### Structural insights into ClpXP function

The ^439^LGF^441^ loop in human ClpX mediates complex assembly by docking into hydrophobic pockets between ClpP subunit pairs (166, 183, 184). The six-to-seven subunit mismatch leaves one of the hydrophobic pockets of ClpP unoccupied, which contributes to substrate translocation (185). As a AAA+ unfoldase from the HCLR clade (53), ClpX harnesses the energy from ATP hydrolysis to exert a pulling force that unravels and translocates substrates into ClpP (186, 187). Structures of bacterial (137, 185, 188) and most recently human ClpXP published by the Lander group (38) have helped elucidate this mechanism.

**Figure 3:**
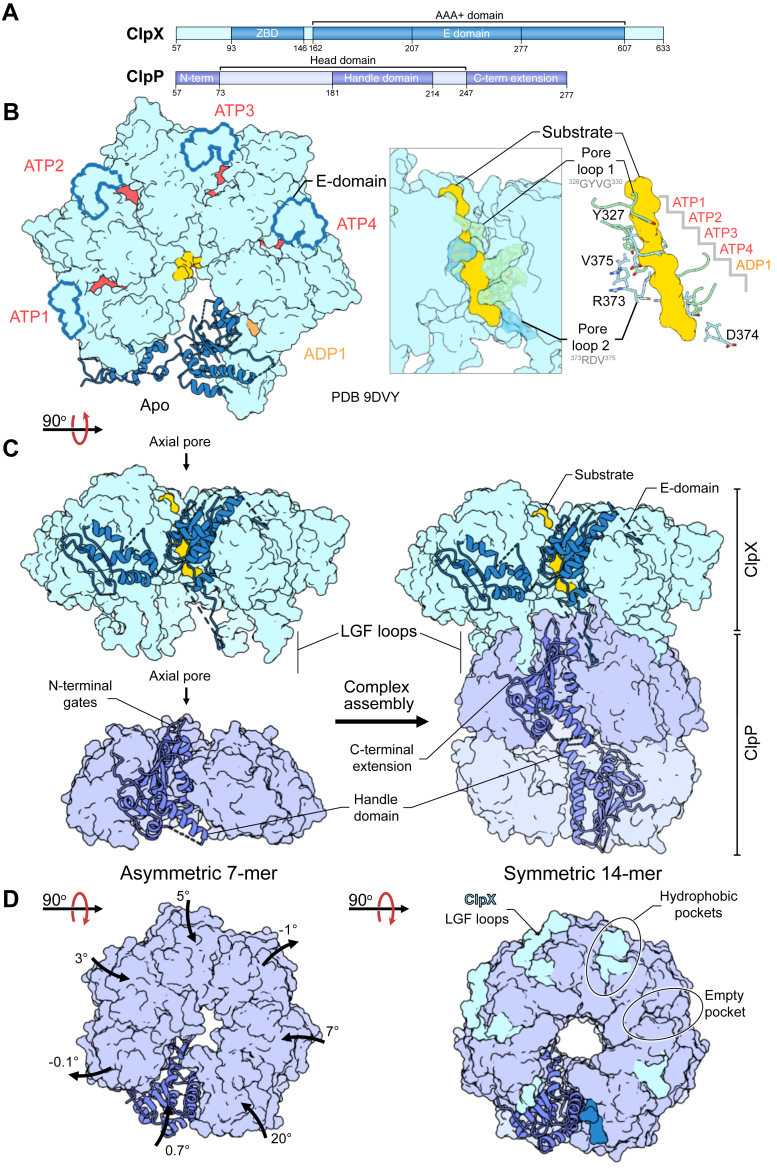
**Structural insights into ClpXP function**. *A*, upon import into the mitochondrial matrix, an MTS (residues 1–56) is cleaved to generate the mature forms of the ClpX unfoldase and the ClpP protease; *B*, ClpX assembles into a hexameric ring with nucleotide binding sites at the inter-subunit interfaces of the AAA+ domains. Human ClpX features a eukaryotic-specific insertion, the E-domain (residues 207–277), that extends across the nucleotide-binding pocket and engages the adjacent subunit. Sequential ATP hydrolysis regulates substrate unfolding and translocation into the ClpP protease. Pore loop 1 and 2 side chains intercalate into the substrate backbone in a nucleotide-bound state, adopting a spiral staircase arrangement in which the pore loop residues encircle the substrate; *C*, complex assembly is mediated by LGF loop motifs on ClpX, which dock into inter-subunit hydrophobic pockets of ClpP. Human ClpP forms gated heptameric rings, which upon ClpX binding, dimerize *via* handle domain interactions to form a tetradecameric proteolytic chamber that adopts the extended conformation upon active site engagement by substrates; *D*, the asymmetric ClpP heptamer shifts to a symmetric arrangement in response to ClpX binding, with the six-to-seven subunit mismatch maintaining one of the hydrophobic pockets unoccupied.

The N-terminal domain of human ClpX contains a conserved C4-type zinc-binding domain (ZBD; Fig. 3*A*) (189, 190) that mediates POLDIP2 binding and substrate recruitment (177, 191). The ClpX ZBD is followed by its AAA+ domain, housing the Walker-A (^293^GPTGSGKT^300^) and -B (^354^IVFLDE^359^) motifs (192). Human ClpX features a eukaryotic-specific insertion named the E-domain (residues 207–277) that extends from the large AAA+ domain across the nucleotide-binding pocket to engage the neighbouring subunit (Fig. 3*B*) (38, 193). Deletion of the E-domain prevents hexameric assembly, while substitution of a nucleotide-interacting residue (E285A) significantly reduces proteolytic activity. This suggests that the E-domain stabilizes ClpX assembly and is essential for sensing nucleotide state across subunits during substrate translocation. The unfolding function of ClpX relies on sequential ATP hydrolysis and a series of conserved pore loops. At the axial pore, the “RKL” loops are involved in substrate recognition (38, 174). The RKL loops are important for engaging phosphorylated serine or threonine residues on damaged or degraded substrates (174). The pore 1 (^326^GYVG^330^) and pore 2 (^373^RDV^375^) loops line the axial channel and engage the translocating substrate in a nucleotide-bound state (Fig. 3*B*) (38, 185). The bulky aromatic and hydrophobic side chains intercalate into the polypeptide backbone to facilitate translocation and unfolding in response to ATP hydrolysis (38, 185). With this mechanism, ClpX adopts a spiral-staircase arrangement in which the pore loop residues encircle the substrate (Fig. 3*B*). The extent of each subunit’s contact with the incoming substrate is determined by the respective nucleotide state. ATPase-driven motions also coordinate repositioning of the ClpX LGF loops in the hydrophobic pockets of ClpP and pore loop 2 interactions with the axial N-terminal residues of ClpP (38, 185). These contacts maintain chaperone-protease communication, ensuring the processive degradation of the substrate (188, 194).

While the coordinated motions of ClpX enable substrate unfolding and translocation, conformational dynamics in ClpP regulate proteolysis (134, 195). Human ClpP adopts two distinct conformations, an active extended state (38, 196, 197) and an inactive compact state (153, 197). These are also observed in bacterial homologues, which adopt an additional inactive compressed conformation (198). The conformations of the N-terminal gates and the handle domains influence the height of the ClpP barrel and distinguish these states (199, 200). The N-terminal gates are dynamic loops that adopt β-hairpin conformations to form the axial pore to the chamber (196, 201). The handle domains consist of a strand-turn-helix motif and mediate the interlocking of the heptameric rings (Fig. 3*C*) (196). The proximity of the catalytic triad to the handle domain links ClpP function to the adopted conformation (202). Only the extended state has the catalytic triad orientation required for activity (203). Compact and compressed states characterized by a shortened or kinked handle helix, respectively, and a disordered handle β-strand, misalign the catalytic triad, thereby rendering ClpP inactive (203). Human ClpP includes a unique 28-residue C-terminal extension not found in bacterial homologues. This unstructured C terminus is thought to occupy the hydrophobic pockets of ClpP and negatively regulate ClpX binding, given that the deletion of this extension promotes ClpXP interaction and substrate degradation (38, 163). Human ClpP exists in an equilibrium of heptameric and tetradecameric assemblies (134). In solution, human ClpP is predominantly heptameric with asymmetric subunit organization and an inactive compact conformation (Fig. 3*D*) (197). ClpX binding to the apical hydrophobic pockets of ClpP shifts the equilibrium toward a compact tetradecameric population with symmetric ring arrangement (Fig. 3*D*) (38). The active extended state, however, is only populated once the substrate has occupied the active-site (38).

The conformational plasticity of human ClpXP provides multiple opportunities for modulation by small molecules, either through orthosteric or allosteric mechanisms. Currently available α-aminoboronic orthosteric inhibitors lack selectivity for human ClpP (204). Some bacterial active-site inhibitors also target human ClpP activity, including β-lactones (148) and phenyl esters (205) (Table 2). However, because of the protease’s inherent conformational dynamics, orthosteric inhibitor binding does not always result in suppression of activity. At sub-stoichiometric concentrations, orthosteric inhibitors have been reported to paradoxically activate ClpP – a phenomenon known as hormesis (206, 207). The occupation of the active-sites by peptidomimetic compounds triggers the extended, proteolytically active conformation (38, 197, 208, 209). This activation also stabilizes the N-terminal β-hairpins, highlighting an allosteric network between the handle domain and the N-terminal gates (197, 210). Mimicking ClpX interactions to open the N-terminal gates is another strategy to disrupt ClpP activity. ClpP allosteric activators that bind the apical hydrophobic pockets, displace ClpX and induce dysregulated protein degradation have been the focus of extensive development and optimization (211).

Dordaviprone (ONC201), a potent imipridone-class ClpP activator, was first identified in a compound screen against human colorectal cancer (212). Although initially thought to function as an antagonist of the dopamine D2-like receptor (213, 214), more recent work identified ClpP as its primary molecular target (153, 215). In preclinical studies (216, 217, 218), Dordaviprone demonstrates efficacy in hematologic and solid tumors *in vitro* and *in vivo*. Once ClpP was established as the molecular target for Dordaviprone (215, 219), several derivatives with optimized scaffolds and improved potency have been developed (220, 221, 222, 223). Bacterial ClpP activators known as acyldepsipeptides (ADEPs) have also been adapted to target the human homologue (Table 2) (224). Other human ClpP-specific activators include molecules D9 (225) and ZK53 (Table 2) (226). Binding of these to the hydrophobic pockets of ClpP promotes N-terminal hydrophobic contacts that drive β-hairpin formation and stabilize the axial pore (201, 224). Motivated by the role of the complex in human health, these developments aim to modulate ClpXP function for therapeutic intervention.

### Implications of ClpXP in human health and therapeutic potential

Disruption of ClpXP-mediated substrate degradation contributes to human diseases marked by mitochondrial dysfunction (227, 228). Gain- and loss-of-function mutations in mitochondrial ClpP are linked to Perrault syndrome type 3, an autosomal recessive and heterogeneous disorder characterized by hearing loss and undeveloped or absent ovaries (229, 18). To date, seven *CLPP* mutations have been identified that either affect ClpP activity by targeting regions near the active site, such as the handle domain, or prevent ClpX binding by altering hydrophobic pocket residues (230). The mutations associated with the most severe developmental defects are Y229D, which abolishes both ClpXP- and ClpP-mediated degradation, and T145P, which also prevents ClpX binding but significantly activates ClpP activity (230). Another autosomal recessive disease is Friedreich ataxia, in which reduced mitochondrial levels of frataxin, a Fe-S assembly protein, impair ATP production and cause degeneration of spinal cord tissue and cardiomyopathy (231). The upregulation of ClpP and LONP1 in frataxin deficiency, along with ClpXP and LONP1-mediated degradation of mitochondrial Fe-S-containing proteins, suggests that both proteases contribute to the disease (159, 232). Impaired mitochondrial function is a common feature of neurodegenerative diseases (233). In sporadic Parkinson’s disease, ClpXP degrades complex I and mitochondrial kinase PINK1 under oxidative stress, contributing to their deficiency, while ClpP knockdown leads to their accumulation (68, 234). Altered ClpP expression has also been reported in Alzheimer’s disease (235), hereditary spastic paraplegia (236), and Huntington’s disease (237). In tissues with high energy demands such as liver, heart, and skeletal muscle, loss of ClpP suppressed UPR^mt^, reduced mitochondrial respiration and impaired mitochondrial translation (229, 238). Glycolysis is upregulated to compensate for the loss of mitochondrial respiration, and ClpP has also been implicated in responses to high-fat diets and adaptive thermogenesis, indicating a role for the protease in type 2 diabetes (161, 239).

The maintenance of mitochondrial proteostasis by ClpXP is also a recognized therapeutic vulnerability in multiple cancers (240, 241). In hematological cancers such as acute myeloid leukemia, genetic or chemical inhibition of ClpP selectively reduces the viability of cells with high ClpP expression (242). Activation of the protease by Dordaviprone also induces malignant cell death (153, 215). ONC201 is effective in solid tumours as well, including squamous cell, breast, colorectal, and diffuse midline glioma, for which it recently received FDA approval for clinical use in adult and pediatric patients with recurrent H3 K27M–mutant diffuse midline gliomas (217, 226, 243, 244, 245, 246). Interestingly, lymphoma cell lines acquired resistance to ONC201 through a D190A substitution in the N-terminal end of the handle helix (153). D190A inhibits ClpP activity by preventing tetradecameric assembly and activator binding (153). ClpP has also been implicated in ovarian, prostate, liver, cervical, and colorectal cancers, although effects on cell viability vary by cancer type (116, 150, 247, 248, 249, 250). Mitochondrial ROS production is critical for sustaining oncogenesis, and ClpP contributes by targeting damaged mitochondrial respiratory subunits for degradation, such that function dysregulation can selectively suppress malignant growth (153, 251, 252). However, cancer cell sensitivity to ClpP inhibition or activation is dependent on ClpP expression levels (153, 224). Tumors with low ClpP expression are less responsive to ClpXP modulation, highlighting a potential for ClpP as a predictive biomarker for chemotherapy effectiveness (153). Given that chemotherapy-resistant cancer cells are more reliant on OXPHOS compared to healthy cells, modulation of ClpXP activity represents a promising approach to kill drug-resistant cancer cells (249, 253).

## YME1L protease

### YME1L domain organization

**Figure 4:**
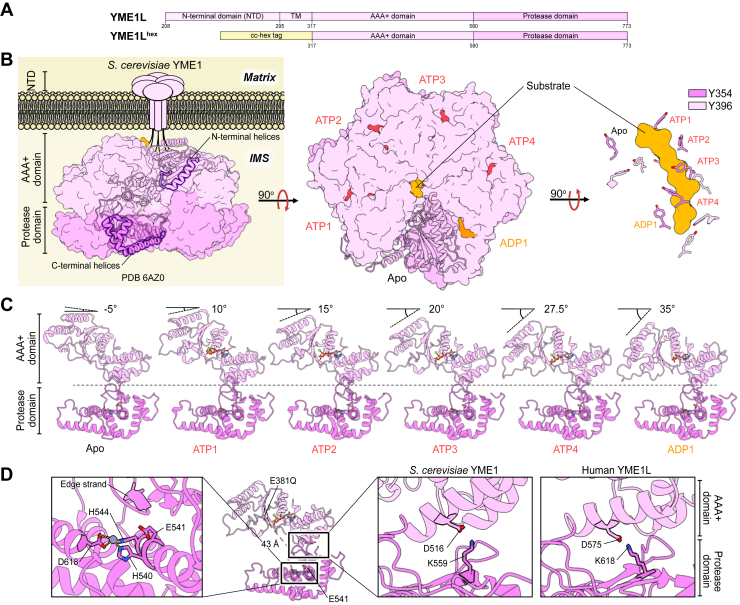
**Structural insights into the YME1L functional mechanism**. *A*, each YME1L polypeptide contains an N-terminal domain (NTD), a transmembrane (TM) region, and two catalytic domains: a AAA+ domain and a protease domain. *In vitro* YME1L studies largely rely on the expression of a soluble synthetic YME1L construct (YME1L^hex^), where a self-associating coiled coil (cc-hex) is expressed *in lieu* of the NTD, and TM regions. *B*, YME1L forms a membrane-bound homohexamer with the catalytic domain oriented toward the IMS. A structure of an ATPase-deficient Walker-B variant of *S*. *cerevisiae* YME1 (^E381Q^YME1^hex^), gives structural insight into the YME1L mechanism. YME1 couples ATP hydrolysis to motions of Y354 and Y396 of pore loops 1 and 2, respectively. *C*, YME1 AAA+ domains adopt a steep helical register relative to one another, a structural feature that defines pore loop 1 and 2 conformation; *D*, substrate entry into the chamber formed by the protease domains prompts cleavage by a Zn^2+^-dependent active site. An E600Q substitution in YME1L (E541 in YME1), significantly accelerates ATPase rates, though the catalytic E439 of the Walker-B motif is 43 Å away from the substituted residue. This allostery is mediated by a conserved salt bridge seen in both the ^E381Q^YME1^hex^ structure and AlphaFold3 (360) predicted structure of YME1L.

The human mitochondrial i-AAA (intermembrane space AAA) protease, hereafter referred to as YME1L (yeast mtDNA escape 1–like), is one of two membrane-anchored members of the AAA+ superfamily localized to the IMM. YME1L belongs to the FtsH family of proteases, a bacterial ancestral ATP-dependent metalloprotease most extensively studied in *E*. *coli* (254, 255). Like most AAA+ enzymes (256), YME1L assembles into a homohexamer to form an active proteolytic complex. Each YME1L polypeptide in the holoenzyme assembly includes a matrix-facing NTD and two IMS-oriented catalytic domains: an AAA+ domain and a M41 metalloprotease domain (Fig. 4*A*) (254, 255). The catalytic domains form a stacked barrel comprised of the hexameric ring of the AAA+ domains proximal to the IMM and a second ring distal to the IMM formed by the protease domains within which the proteolytic active sites rest (Fig. 4*B*) (35, 257). This assembly forms a central pore through which protein substrates are unfolded and translocated into the protease chamber for degradation (Fig. 4*B*) (35, 257). Unlike bacterial FtsH and its mitochondrial matrix-oriented AFG3L2 homologue, YME1L contains only a single transmembrane (TM) helix, bridging the NTD and the AAA+ domain (258). This TM helix has historically hindered *in vitro* functional and structural investigations of YME1L. This has been circumvented by the expression of a hexamerization tag on the N terminus of the AAA+ domain *in lieu* of the NTD and the TM helix (YME1L^hex^; Fig. 4*A*) (35, 259, 260, 261). This tag is composed of a 32-residue *de novo* sequence (coined cc-hex) that is sufficient for coiled-coil-mediated hexamerization (262), followed by a 10-residue linker tethered to the YME1L AAA+ domain (Fig. 4*A*) (259).

### YME1L function

Though the IMS houses only ∼5% of the mitochondrial proteome, IMS proteins are responsible for essential functions including protein import, small-molecule and ion transport, and OXPHOS (263). Turnover of damaged IMS proteins is undertaken by members of the mtPQC system localized in this subcompartment, including the HTRA2 serine protease, as well as the metalloproteases ATP23, mitochondrial oligopeptidase M (MEP), and YME1L (10). As the sole AAA+ protease in the IMS, YME1L supports homeostasis by regulating mitochondrial dynamics (264, 265, 266), membrane lipid (267) and protein (268) import, and quality control of respiratory chain subunits (269).

YME1L is part of a network of IMM-embedded proteases that reciprocally degrade one another to modulate mitochondrial dynamics. OPA1 is a dynamin-family GTPase that promotes mitochondrial fission upon processing into its short isoform (S-OPA1), and fusion when retained in its long isoform (L-OPA1) (7, 270). OMA1, a stress-activated ATP-independent metalloprotease, and YME1L both cleave OPA1 at two distinct sites, S1 and S2, respectively (Fig. 1) (271, 272). Under normal conditions, YME1L processes a subset- of L-OPA1 at S2 to maintain a balanced pool of isoforms and to preserve cristae integrity (264, 273). In this steady state, YME1L degrades OMA1, preventing excessive OPA1 processing (274, 275). Conversely, under sustained stress conditions where ATP levels fall, OMA1 degrades YME1L and generates excess S-OPA1, initiating mitochondrial fragmentation and apoptosis (7, 264, 275, 276). Studies of *S*. *cerevisiae* YME1, the closely related YME1L homologue (42% sequence identity), suggest YME1L may also play a key role in the initiation of mitophagy. YME1 degrades the IMS-oriented C terminus of Atg32, an OMM-embedded protein that mediates recognition of mitochondria by the autophagy machinery on its cytosolic interface (9). Processing of the Atg32 C terminus by YME1 recruits a cytosolic scaffold protein, Atg11, to the OMM, propagating the mitophagy signaling cascade (9). YME1L also regulates cardiolipin levels in the IMM, an attribute which is inextricably linked to mitochondrial dynamics. YME1L degradation of PRELID1, a phospholipid carrier protein that facilities import of the cardiolipin precursor phosphatidic acid, renders cells susceptible to apoptosis (Fig. 1) (277). YME1L also supports attenuation of mitochondrial protein import under cellular stress (268). YME1L selectively degrades the stress-response subunit, TIM17A, of the larger translocase of the inner membrane 23 (TIM23) protein import complex, obstructing protein import (Fig. 1) (268). Beyond its roles in processing specific substrates to regulate organellular function, YME1L also plays a broader “housekeeping” role by clearing the IMS of damaged and misfolded proteins (269, 278, 279). YME1L maintains the integrity of respiratory chain subunits through degradation of oxidatively damaged or unassembled respiratory subunits, NDUFB6, COX4, COX2, and ND1 (269, 280, 281). Additionally, functional studies of *S*. *cerevisiae* YME1 shows its role in clearing misfolded members of the small translocase of the inner membrane (TIM) family, namely Tim10 (278, 279). Improper disulfide bond formation within the Tim9/10 protein import complex, often induced by oxidative stress, prompts Tim10 degradation by YME1 in the IMS (278, 279). YME1-mediated degradation of the human Tim10 homologue, TIMM13, suggests this role is retained in YME1L (282).

Despite characterization of numerous substrates, strict rules governing YME1L substrate recognition remain elusive. Studies of FtsH substrate engagement first revealed a preference for unstructured N- or C-terminal degrons within membrane proteins, typically 10 or 20 residues in length, respectively (283, 284). Later investigations of YME1L corroborated this, finding a preference for terminal, unstructured sequence-specific degrons of at least five amino acids in length (259). Fusion of the well-characterized 20-residue AAA+ degron, β20 (QLRSLNGEWRFAWFPAPEAV), to the termini of model proteins rapidly increases degradation by YME1L (72, 259). Conversely, degradation rates fall sharply when this same degron is placed within the substrate (259). Inclusion of a FhhF (F: phenylalanine; h: any hydrophobic residue) sequence, a motif found within β20, in model substrates accelerates degradation (259). The relative ubiquity of this sequence within characterized YME1L substrates lends credence to its identity as a degron. This includes PRELID1 and OMA1, as well as 21 other proteins of the 127 total proteins found in the IMS proteome (259). It is likely that other YME1L substrates exist within the IMS beyond those explicitly described in the literature. Indeed, numerous other constituents of the IMS proteome contain this FhhF degron, though they are not explicitly classified as YME1L substrates (259). For example, experiments identifying YME1L substrates using a AAA+^TRAP^ approach (285) identified respiratory chain complex I subunits, ND2, ND5, and ND6 as likely YME1L substrates (269). Of these three subunits, ND5 and ND6 also contain the FhhF motif, further supporting their identity as likely YME1L substrates. The lack of this degron in ND2 also suggests that other sequences rather than just this motif facilitate YME1L-mediated degradation. Indeed, model protein substrates devoid of this motif are degraded *in vitro* (259). Others have suggested the lack of strict YME1L substrate recognition rules and a preference for hydrophobic residues is characteristic of a single IMS AAA+ protease responsible for surveilling a diverse proteome susceptible to damage and misfolding (258, 260).

Current understandings of substrate engagement rely on mutational studies of YME1 (286). It has been proposed that misfolded substrates are engaged by the conserved solvent-exposed N-terminal helices (NH) of the YME1 AAA+ domain (YME1L: 338–360) or alternatively the C-terminal helices (CH) of the protease domain (YME1L: 708–767; Fig. 4*B*) (286) to increase local substrate concentrations at the apical pore (258). Likewise, it is thought that leucine residues within the CH of FtsH mediate substrate engagement (257). A high degree of a conservation of these residues in YME1L supports this supposed engagement mechanism. Substrates vary in their dependence on these NH and CH regions for degradation, with membrane-embedded proteins appearing less reliant on the more distal CH helices (286). However, the high degree of local charge within these helical regions is somewhat incongruent with the hydrophobic motifs that are thought to mark substrates for degradation, necessitating additional mechanistic insight. Following engagement of IMM-embedded substrates, ATP-powered threading by the AAA+ domain is believed to facilitate the entropically unfavourable extraction of membrane proteins from the hydrophobic lipid bilayer (258). Alternative models suggest that the non-catalytic YME1L TM helices disrupt IMM-bound protein-lipid contacts, lowering the energetic cost of membrane-embedded protein extraction (258).

### Structural insights into YME1L function

Once engaged, substrates are processively unfolded by the AAA+ domain and threaded into the interior of the proteolytic chamber for proteolysis. The mechanism underlying protein unfolding and translocation follows the sequential hand-over-hand mechanism as described above (Fig. 2*C*) (35, 96, 186, 257, 287). Similar to other AAA+ motors, YME1L depends on cooperativity between adjacent AAA+ domains for sequential ATP hydrolysis (32, 261). Molecular-level insights into YME1L around-the-ring ATP hydrolysis and substrate degradation have relied on cryo-EM investigations of an ATPase-deficient Walker-B variant (E381Q) of *S*. *cerevisiae* YME1, in which the catalytic domains were expressed with an N-terminal hexamerization tag (^E381Q^YME1^hex^) (35). The structure of ^E381Q^YME1^hex^ revealed that two conserved arginine fingers (YME1L: R494 and R497) act in trans to sense the nucleotide state of the neighboring subunits, contacting the ATP γ-phosphate occupying the adjacent subunit (35, 96, 288, 289). An ISS motif (YME1L: ^468^DGF^470^) communicates nucleotide state between adjacent subunits (35, 290). Like cryo-EM structures of other AAA+ proteases (36, 37, 57, 291, 292), structural analysis of ^E381Q^YME1^hex^ reveals an actively translocating substrate modeled in the central pore, revealing additional mechanistic insight. Central channel-facing aromatic residues of pore loops 1 and 2, Y354 and Y396, respectively, intercalate between substrate side chains, directly contacting the polypeptide backbone (35, 287, 293). During translocation, YME1 forms a spiral staircase conformation, with the tilt of each AAA+ subunit increasing by 5 to 7.5° relative its associated protease domain (Fig. 4*C*) (35). Four of the six ^E381Q^YME1^hex^ subunits that retain ATP occupy the four highest positions in the staircase (positions ATP1-ATP4; Fig. 4*B*). The sole subunit that retains ADP occupies the lowest pitch while the sixth subunit acts as a nucleotide-free “step” subunit, bridging the lowest and highest subunits (Fig. 4*B*) (35). Conformation of pore loop residues Y354 and Y396 mirror the spiral staircase conformation of each of the YME1 AAA+ subunits (Fig. 4*B*) (35). Pore loop tyrosine residues of ATP-bound subunits occupy the highest helical pitch and make direct contact with the substrate backbone, while pore loop tyrosine-substrate contacts of ADP-bound subunits are weaker (35). Pore loops of apo subunits remain entirely disengaged from the translocating substrate. Like the other mitochondrial AAA+ proteases discussed, as individual YME1 AAA+ domains cycle from ATP1 position to the ATP4 position, undergo hydrolysis, and release ADP, the pore loops sample the conformations defined by the nucleotide state of its associated subunit to pull the substrate into the proteolytic chamber (Fig. 2*C*) (32).

Entry of unfolded polypeptides into the proteolytic chamber prompts peptide bond cleavage by the Zn^2+^-dependent protease domain. The YME1L protease domain houses the proteolytic active site comprised of the canonical HExxH metal-binding motif characteristic of the M41 metalloprotease family to which it belongs (Fig. 4*D*) (35, 294, 295, 296). A Zn^2+^ ion is coordinated by the two histidine residues of this motif and an additional aspartate residue of the protease domain (Fig. 4*D*) (294). Despite residing in close proximity to the AAA+ domain, the protease domain maintains C6 planar symmetry irrespective of the AAA+ motor’s functional cycle (35). A flexible linker conserved across bacterial homologues, including FtsH, connects the AAA+ and protease domains and effectively shields the protease ring from large-scale structural rearrangements driven by ATP hydrolysis (35, 296). Nevertheless, allosteric communication does exist between these adjacent catalytic domains. Indeed, substitution of the catalytic glutamate of the YME1L protease domain (^E600Q^YME1L) induces significant changes in the structural dynamics of the AAA+ domain, as revealed by hydrogen-deuterium exchange mass spectrometry (HDX-MS) (261). Though this substitution abolishes YME1L protease activity, ^E600Q^YME1L ATPase rates are enhanced 8.6-fold relative to wild-type YME1L, a fact made especially noteworthy given the 43 Å distance between the AAA+ and protease domain active sites (Fig. 4*D*). Amino acid substitutions revealed that this activation is propagated through an interdomain salt bridge formed between D575 and K618 of the AAA+ and protease domains, respectively (Fig. 4*D*) (261). The ^E381Q^YME1^hex^ structure shows structural conservation of this salt bridge, further highlighting its importance for YME1L function (Fig. 4*D*). Structural studies of FtsH reveal that nucleotide binding induces subtle structural changes in a conserved so-called “edge β-strand” above the protease active site to regulate substrate access (Fig. 4*D*) (296). HDX-MS studies point to conservation of this allosteric regulation in YME1L, as peptides spanning this β-strand exhibit reduced D-uptake upon nucleotide binding, consistent with an allosteric structural transition (261).

### Implications of YME1L in human health and therapeutic potential

A deleterious missense *YME1L* mutation that encodes R206W YME1L (^R206W^YME1L) causes optic atrophy 11, a recessive disease associated with progressive vision loss (297). This missense mutation, located in the MTS immediately preceding the NTD, obstructs productive MTS cleavage by the mitochondrial processing peptidase, MPP, induces accumulation of the inactive precursor polypeptide and prevents efficient maturation of functional YME1L (297). ^R206W^YME1L expression in fibroblasts manifests in impaired OPA1 cleavage, mitochondrial fragmentation, and slowed cell growth (297). This is consistent with the hallmarks of YME1L knockdown, where altered cristae morphology and increased fragmentation are observed due to dysregulated OPA1 processing (269). Similarly, mice with *YME1L*-deficient nervous systems show degeneration in spinal cord axons and develop microphthalmia with cataracts (298).

Human YME1L proteolysis helps reshape the mitochondrial proteome in response to hypoxic and nutrient deprivation stresses characterized in pancreatic cancer cells (299). YME1L overexpression has been documented in various types of cancer and is thought to promote cancer cell survival under conditions that damage the mitoproteome (15, 300, 301). For example, YME1L overexpression has been noted in lung cancer, osteosarcoma, and glioma cells (15, 300, 301). Critically, silencing of YME1L in these cell lines induces mitochondrial dysfunction, depolarization, and apoptosis (15, 300, 301). This highlights the extent to which YME1L is a dependency in several different cancer types, owing in large part due to its diverse roles related to mitochondrial biology. With implications in numerous cancer types, the therapeutic potential of targeting YME1L is rapidly emerging. To our knowledge, no YME1L inhibitors have been characterized to date, complicating efforts to explore YME1L as a therapeutic target. Further investigations focused on the development of YME1L-specific effector molecules will be foundational in understanding how YME1L can be dysregulated to disrupt disease.

## m-AAA

### m-AAA domain organization and biological function

**Figure 5:**
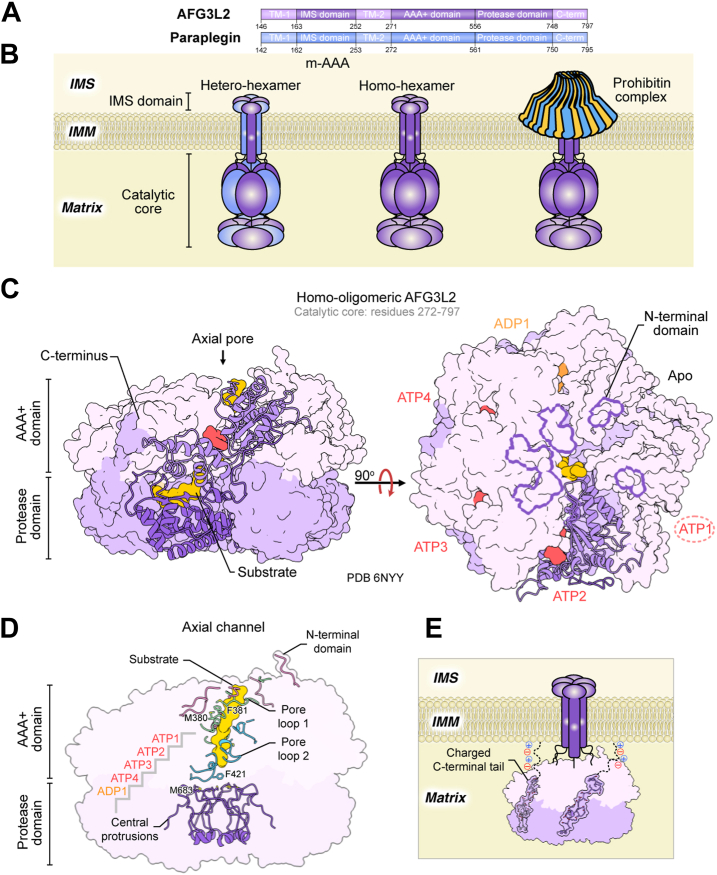
**Structural insights into m-AAA function**. *A*, human m-AAA protease is formed by AFG3L2 and paraplegin, both consisting of an N-terminal domain (NTD) and two transmembrane regions tethered to the IMM with AAA+ and zinc-metalloprotease domains facing the mitochondrial matrix. *B*, m-AAA assembles as homohexameric AFG3L2 or heterohexamers of alternating AFG3L2 and paraplegin subunits. Regulation of m-AAA activity is associated with membrane scaffold proteins, prohibitins, which form large complexes that encapsulate m-AAA and govern substrate entry; *C*, the substrate-bound human homo-oligomeric m-AAA structure was determined using a truncated AFG3L2 construct (residues 271–797), which only retains the catalytic core. This structure reveals the nucleotide state for 4 out 6 subunits, sufficient to clarify the conserved hand-over-hand substrate translocation mechanism. *D*, AFG3L2 displays central channel features, distinct from those of YME1L, that modulate substrate engagement. NTD extensions form a spiral staircase arrangement on the apical surface of the AAA+ domains, resembling the organization of the central pore loop 1 and 2, which together encircle the substrate. Pore loop 2 is further involved in contacts with central protrusions in the protease domain which direct incoming substrates to proteolytic active sites. *E*, AFG3L2 also contains a highly charged, unique C terminus tail that extends from the base of the protease domain to the protease and AAA+ domains of neighbouring subunits, oriented toward the membrane-proximal surface.

Similar to YME1L, m-AAA shares high sequence similarity to prokaryotic FtsH protease, consisting of an NTD and 2 TM regions tethered to the IMM with AAA+ and zinc-metalloprotease domains facing the mitochondrial matrix (Fig. 5*A*) (255, 302). Human m-AAA protease is a hexamer consisting of either six identical AFG3L2 subunits or heterohexamers of alternating AFG3L2 and paraplegin subunits (Fig. 5*B*) (303, 304, 305). AFG3L2 and paraplegin share 49% sequence identity and 58% sequence similarity (294). A third m-AAA subunit, AFG3L1, is expressed in mice, where it forms homo- or hetero-oligomeric assemblies with paraplegin (306). The first mitochondrial m-AAA protease subunits identified were yeast homologues Yta10 (or Afg3p) and Yta12 (or Rca1p), proposed to mediate the degradation of IMM-associated proteins (307, 308). Loss of proteolytic activity in these subunits disrupts mitochondrial translation and leads to defective assembly of respiratory chain and ATP synthase subunits (309, 310, 311). These roles are conserved in mammalian homologues, including in mouse and human, with the targeting of certain substrates preserved across species (302, 312).

In both yeast and mice, m-AAA processes the nuclear-encoded ribosomal protein MrpL32 upon mitochondrial import (Fig. 1) (313, 314, 315). In the absence of m-AAA, translation of respiratory chain subunits is disrupted, resulting in defective complex assembly and impaired oxidative phosphorylation (313). Yeast m-AAA also mediates the maturation of the ROS-scavenging cytochrome *c* peroxidase (ccp1) together with rhomboid proteases in the IMM (316). ATP hydrolysis by m-AAA drives the dislocation of Ccp1 from the membrane, positioning the protein for intramembrane cleavage (317). This cleavage is primarily affected by substitutions in the Walker-A and -B motifs of the AAA+ domain, rather than substitutions in the protease domain’s zinc-binding site, potentially highlighting an isolated function of the AAA+ domain (317). Human mitochondrial ATP production is also indirectly influenced by m-AAA *via* regulation of Ca^2+^ homeostasis (318). Ca^2+^ uptake is mediated by the IMM Ca^2+^ uniporter (MCU) channel, with the subunit EMRE facilitating permeation (319). Increases in matrix Ca^2+^ levels enhances ATP output (320). m-AAA targets excess or unassembled EMRE subunits, ensuring proper channel assembly and function (Fig. 1) (319). This regulation is particularly critical in neuronal mitochondria, where the loss of m-AAA leads to accumulation of constitutively active channels and mitochondrial Ca^2+^ overload (321). m-AAA has also been implicated in the regulation of mitochondrial morphology through processing of the IMM GTPase OPA1 and IMM protease OMA1 (7, 322, 323, 324, 325, 326). Interestingly, the extent to which AFG3L2 or paraplegin subunits mediate OPA1 processing is tissue-specific (322, 327). This suggests that substrate specificity may differ between homo- and hetero-oligomeric m-AAA isozymes, although definitive evidence is lacking (303). Regulation of m-AAA activity can also be mediated by the association with prohibitins (PHB-1 and -2) (328). PHBs are evolutionarily conserved membrane scaffold proteins that form high-molecular weight ring complexes, predominantly found in the mitochondrial IMM (329, 330). Yeast mitochondria lacking PHB-1 or -2 exhibited increased m-AAA-mediated degradation of non-assembled IMM proteins (328). PHB interacts with the NTDs of m-AAA to form a cage-like complex that modulates access of membrane-embedded substrates to degradation (328, 331, 332).

### Structure and functional mechanism

The cryo-EM structure of full-length Yta10 and Yta12, co-expressed in yeast cells, provided the first structural insights into the mechanism of the mitochondrial m-AAA protease (333). This 12 Å-resolution structure reveals a hexameric bipartite assembly ∼137 Å in height. Six N-terminal and 12 TM domains form a funnel-like membrane anchor for the AAA+ and protease domains, which together enclose the proteolytic chamber (333). More mechanistic details were revealed by the substrate-bound human homo-oligomeric m-AAA structure determined at 3.1 Å using a truncated AFG3L2 construct (residues 272–797) that excludes the N-terminal and TM domains, retaining only the catalytic core (36). This structure showed that AFG3L2 assembles into two stacked rings, with an asymmetric AAA+ spiral above a symmetric protease base (Fig. 5*C*) (36). Density for the peptide substrate threads through the center of the AAA+ domain and extends into two protease active sites (36). While the fundamental mechanism of substrate degradation is conserved between m-AAA and YME1L, these structures uncover unique features that distinguish the two complexes.

The N-terminal IMS and TM domains of m-AAA, although dispensable for complex assembly and proteolytic activity, are essential for the degradation of integral membrane proteins *in vivo* (315, 334). These domains are proposed to function as a gated pore that dislocate integral substrates and mediate their translocation across the mitochondrial inner membrane into the proteolytic chamber (186, 317, 335). The N-terminal IMS domains are also predicted to interact with prohibitins *via* a disordered SPFH-interacting motif (SIM) exposed in the IMS (331). Prohibitins thus encapsulate m-AAA and regulate substrate entry Fig. 5*B*) (336). The N terminus of the AAA+ domain contains a flexible glycine-rich region followed by ordered extensions that link the TM segments to the catalytic core (36). In the human AFG3L2 structure, these extensions form a spiral staircase above the AAA+ domains, directly contacting the substrate and mirroring the organization of the pore loops (Fig. 5*D*) (36). Mutagenesis of key residues in this region (*e*.*g*., F289 and L299) reduces proteolytic activity without affecting ATP hydrolysis, consistent with the N terminus stabilizing substrate engagement and processive unfolding (36).

The classic AAA+ hand-over-hand mechanism, in which sequential ATP hydrolysis drives substrate translocation through the central pore, is conserved in mitochondrial m-AAA (337). Like YME1L, AFG3L2 coordinates the inter-subunit signaling motif conformation and pore-loop positioning with the nucleotide state, thereby allosterically regulating substrate translocation (35, 36). However, AFG3L2 displays unique pore-loop features that modulate substrate engagement (186). In pore loop 1, M380 preceding the conserved F381 forms inter-subunit contacts that encircle the substrate (36). Pore loop 2 contains a four-residue insertion that positions the central F421 proximal to the substrate, forming a second spiral staircase that engages the translocating polypeptide, an arrangement unique among the AAA+ family (Fig. 5*D*) (36, 186). These interactions strengthen substrate engagement and facilitate efficient processing. The pore loop 2 residue F421 from the lowest spiral subunit contacts the M683 residue of the central protease protrusions, which consist of upward-projecting loops (Fig. 5*D*) (36). The substitution of either F421 or M683 specifically impairs substrate degradation, highlighting a regulatory element between the AAA+ and protease domains in directing incoming substrates (36). The C terminus of AFG3L2 extends from the base of the protease domain engaging the protease and AAA+ domains of adjacent subunits, orienting a highly charged tail towards the membrane-proximal surface (36). These inter-subunit interactions stabilize the complex assembly, as deletion of the C-terminal region beyond residue 750 disrupts both ATPase activity and substrate degradation.

### Implications of m-AAA in human health and therapeutic potential

Genetic alterations in the m-AAA protease subunits paraplegin and AFG3L2 have been implicated in neurodegenerative diseases (318). An autosomal recessive form of hereditary spastic paraplegia (HSP) characterized by degeneration of corticospinal motor axons is caused by diverse substitutions in paraplegin (304, 338, 339, 340, 341). Loss of paraplegin manifests as compromised mitochondrial respiration, accumulation of damaged mtDNA and abnormal mitochondrial morphology, as demonstrated in yeast complementation studies and mouse models (342, 343, 344, 345). Most disease-associated mutations occur in the protease domain of paraplegin and disrupt both proteolytic and ATPase functions, whereas some ATPase-impaired variants can retain function through assembly with AFG3L2 (343, 346). Paraplegin's role in HSP has been proposed to stem from altered m-AAA assembly rather than complete loss of the complex, as subunit composition is tissue-specific (303). Paraplegin deficiency also promotes the formation of AFG3L2 homo-oligomers, which may modify substrate specificity (318). Paraplegin substitutions have also been linked to chronic ophthalmoplegia resulting from defects in mtDNA maintenance (347).

AFG3L2 substitutions are the cause of three neurological diseases; spinocerebellar ataxia type 28 (SCA28) (35, 335, 336, 337), spastic ataxia type 5 (SPAX5) (348) and dominant optic atrophy type 12 (DOA12) (349, 350). In DOA, the R468C substitution at the AAA+ inter-subunit interface disrupts the Arg-fingers essential for ATP-binding, impairing complex assembly and thereby inhibiting AFG3L2 ATPase and proteolytic activity (36). Most disease-associated variants are associated with SCA28, a progressive degeneration of the cerebellum and spinal cord that results in impaired muscle coordination and balance (351). Studies on yeast and patient-derived cells indicate that these substitutions do not generally destabilize the protein or prevent complex assembly but instead impair ATPase and proteolytic activities (21). Disease-linked AFG3L2 substitutions cluster in four regions of the enzyme: the AAA+ inter-subunit interface near the nucleotide-binding pocket, the lateral helices of the protease domain, residues surrounding the catalytic triad, and the central protrusions at the base of the protease domain (36). Notably, the N432T substitution in the AAA+ domain and E691K in the proteolytic domain act in a dominant negative manner, disrupting central pore structures critical for substrate recognition, unfolding and translocation (21, 36). The remaining SCA28-associated substitutions localize to the central protease protrusions, including M666R, which completely abolishes complex assembly and is associated with one of the most severe patient phenotypes (36).

### Concluding remarks

Structural biology and quantitative biophysics of mitochondrial AAA+ proteases now link nucleotide cycles, movements of the pore loops, and protease gating to phenotypes ranging from neurodevelopmental syndromes to malignancy. Since certain disorders depend on mtPQC, tuning or blocking these systems could be an effective therapy. This mechanistic grounding can help guide how we interpret patient variants, read proteomic remodeling, and design interventions with precision.

There is a pressing need for reagents that modulate the function of mitochondrial AAA+ proteases. For ClpXP, small-molecule allosteric modulators, exemplified by ONC201 (153, 215), have established a foundation for translational development in this space. We anticipate the development of analogous reagents for LONP1, YME1L, and m-AAA that exploit conserved conformational switches and signaling motifs to restore mtPQC, counteract disease-associated alleles, or leverage cancer vulnerabilities. A complementary path to target these proteases is *de novo* protein design (352). Computationally designed new-to-nature binders can be engineered to bind specific interfaces with nanomolar affinity and antibody-like selectivity. These protein-based reagents can serve as mechanistic probes and, with appropriate delivery (353, 354) and safety engineering, as therapeutics that compress discovery timelines beyond what is typical for small molecules. In parallel, targeted protein degradation is expanding the toolkit. Conventional targeted protein degradation relies on the ubiquitin-proteasome or lysosomal systems, and therefore it cannot reach proteins sequestered within the mitochondria. Mitochondrial protease-targeting chimeras address this gap by coupling a ClpP-binding warhead to a ligand for the targeted protein, thereby bringing the substrate to ClpP while stimulating its proteolysis (355). Collectively, these strategies chart a path to precise control of mtPQC and to therapies grounded in mechanism.

Foundational principles established by Alfred Goldberg (356, 357), honored in this *JBC* thematic review series, that ATP-dependent proteolysis is a regulated, energy-coupled, and compartmentalized system for selective protein turnover, anchor the advances reviewed here. By defining how energy consumption, substrate selection, and gated proteolytic chambers confer spatial and temporal control over protein degradation, his pioneering work provided the conceptual framework that now guides mechanistic investigations of mitochondrial AAA+ proteases and informs emerging strategies to modulate their function from mechanism to therapy.

## Conflict of interest

The authors declare the following financial interests/personal relationships which may be considered as potential competing interests: S. V. is an Editorial Board Member for JBC and was not involved in the editorial review or the decision to publish this article. A. D. S. has received research funding from Takeda Pharmaceuticals, BMS, and Medivir AB, and consulting fees/honorarium from Takeda, Astra-Zeneca, BMS, and Novartis Pharmaceuticals. A. D. S. is named on a patent application for the use of DNT cells to treat AML. A. D. S. is on the medical and scientific board of the Leukemia and Lymphoma Society of Canada.
